# Heparin reversal with protamine sulfate after Percutaneous Hepatic Perfusion (PHP): is less more?

**DOI:** 10.1186/s40644-023-00590-7

**Published:** 2023-07-14

**Authors:** Nadia Facchetti, Jan B. Hinrichs, Lena S. Becker, Martin A. Schneider, Roland Brüning, Jan Rademacher, Jochen Lenz, Kirsten Kudrass, Arndt Vogel, Frank K. Wacker, Cornelia L. A. Dewald

**Affiliations:** 1 Institute for Diagnostic and Interventional Radiology, Hannover Medical School, Hannover, Germany; 2 Department of Radiology and Neuroradiology, Asklepios Clinic Hamburg-Barmbek, Hamburg, Germany; 3 Department of Anesthesiology, Asklepios Clinic Hamburg-Barmbek, Hamburg, Germany; 4 Department of Anesthesiology, Hannover Medical School, Hannover, Germany; 5 Department of Gastroenterology, Hepatology and Endocrinology, Hannover Medical School, Hannover, Germany

**Keywords:** Protamine sulfate, Heparin neutralization, Chemosaturation, Percutaneous hepatic perfusion, Thromboembolism, Periprocedural safety

## Abstract

**Purpose:**

Percutaneous hepatic perfusion (PHP) is a palliative intraarterial therapy for unresectable hepatic malignancies. During PHP, high-dose melphalan is infused via the hepatic artery to saturate tumor in the liver with the chemotherapeutic substance. The venous hepatic blood is filtered by an extracorporeal melphalan specific filtration system. Blood clotting in the extracorporeal filter system is prevented by administering unfractionated heparin (UFH) in high doses, which might be reversed with protamine sulfate after the procedure. Aim of this retrospective two-center-study was to analyze the potential effect of UFH reversal with protamine sulfate on complication rates following PHP.

**Materials and methods:**

All patients receiving PHP treatment between 10/2014 and 04/2021 were classified according to their intraprocedural coagulation management: 92 patients/192 PHP received full UFH reversal with protamine (group_PROTAMINE_); 13 patients/21 PHP in group_REDUCED_PROTAMINE_ received a reduced amount of protamine, and 28 patients/43 PHP did not receive UFH reversal with protamine (group_NO_PROTAMINE_). Periinterventional clinical reports, findings and laboratory values were retrospectively evaluated. Complications and adverse events were classified according to Common Terminology Criteria for Adverse Events (CTCAEv5.0).

**Results:**

Thromboembolic events were recorded after 10 PHP procedures (5%) in group_PROTAMINE_, six of which (3%) were major events (CTCAE grade 3-5). No (0%) thromboembolic events were recorded in group_REDUCED_PROTAMINE_ and group_NO_PROTAMINE_. Hemorrhagic events were registered after 24 PHP (13%) in group_PROTAMINE,_ two of which (1%) were major (CTCAE grade 3-4). In group_REDUCED_PROTAMINE_, only minor bleeding events were recorded, and one major hemorrhagic event was documented in group_NO_PROTAMINE_ (2%). There was a significant difference between the percentage of post-interventional thrombopenia in group_PROTAMINE_ (39%) and group_REDUCED_PROTAMINE_ (14%) versus group_NO_PROTAMINE_ (23%) (*p=*.00024). In group_PROTAMINE_ one patient suffered from a severe anaphylactic shock after the administration of protamine.

**Conclusion:**

Our retrospective study implies that there might be a link between the practice of protamine sulfate administration to reverse the full hemodilutive effect of UFH after PHP and the post-interventional risk of thromboembolic events as well as clinically significant thrombopenia. Our data suggest that the standard use of protamine sulfate after PHP in low-risk patients without clinical signs of active bleeding should be critically re-evaluated.

## Introduction

Chemosaturation with percutaneous hepatic perfusion (PHP) is a minimally invasive therapeutic approach to treat primary or secondary hepatic malignancies. The therapeutic aim of PHP is to control intrahepatic tumor growth, palliate symptoms and extent survival. Effectiveness of this liver directed treatment has been demonstrated in several studies [1–7].

PHP takes advantage of the unique vascular network of the liver: while the liver parenchyma is supplied dually via portal vein and hepatic arteries, liver tumors predominantly obtain their blood supply via the hepatic arteries [8]. During PHP, high-dose melphalan is administered via a catheter in the hepatic artery, providing undiluted chemoperfusion to the diseased liver parenchyma. To avoid systemic side effects from the melphalan-enriched blood, the hepatic segment of the inferior vena cava (IVC) is isolated. For this purpose, a double balloon catheter (Delcath Inc. New York, USA) inserted via the common femoral vein is used to occlude the IVC above and below the junction of the hepatic veins [2, 4, 9–11]. The venous blood from the liver is aspirated through side holes in the double balloon catheter and cleansed of melphalan in a filter interposed in an extracorporeal circulation system. Subsequently, the purified blood is returned to the systematic circulation through a jugular port.

It is substantial for a safe intervention to prevent clotting of the blood during the extracorporeal hemofiltration. Thus, unfractionated heparin (UFH) is administered before initiation of the extracorporeal circuit, commencing with an IV bolus of 400 U/kg body weight. UFH inactivates thrombin and activated factor X (factor Xa) through an antithrombin-dependent mechanism [12, 13]. Because the anticoagulant response to heparin varies among patients, the dosage of heparin and its effect must be closely monitored. Activated clotting time (ACT), introduced by Hattersley in 1966, is an objective clotting test used to evaluate the efficacy of heparin in situations that prohibit time consuming laboratory diagnostics [14]. For PHP, an constant ACT above 400s is mandatory to avoid clotting in the extracorporeal hemofiltration system. Maintaining this ACT level needs strict monitoring during the intervention and requires repetitive UFH bolus injections.

A major risk of extensive hemodilution is hemorrhage of all kinds. Therefore, all patients are screened for risk factors contraindicating extensive anticoagulation (like hemorrhage-prone intracranial metastases). In order to avoid unnecessary risks of hemorrhage following PHP, the manufacturer`s instructions recommend to administer protamine sulfate to antagonize UFH before removal of the large catheters and sheaths [15]. Protamine sulfate forms a complex with heparin; one milligram of protamine sulfate neutralizes approximately 100 U of UFH. Due to the different half-lives of protamine sulfate (approx. 7 min) and UFH (about 90 min), timing and dosage of protamine sulfate administration is challenging [16]. As early as in the 20^th^ century, it became apparent that the appropriate dosing management of protamine is crucial as inadequate protamine dosing can unexpectedly influence patient hemostasis [17–19]. In addition to the obvious procoagulant effect, excessive protamine sulfate dosing can paradoxically result in an increased risk of bleeding [16]. The complex interaction of protamine sulfate and UFH might also potentially trigger thromboembolic events following PHP [20]. Reports concerning infrequent and occasionally severe thromboembolic events following PHP have been described in the literature [1, 3, 21], all these studies used protamine as antidote to UFH.

The two centers contributing to this study initially used full dose protamine sulfate to reverse the heparin effect at the end of the PHP procedure according to standard procedure. Due to isolated but severe cardiovascular events, both centers modified the periprocedural protamine management.

The aim of this retrospective study was to analyze the potential effect of protamine sulfate on complication rates following PHP with a particular focus on thromboembolic events and bleeding complications.

## Materials and methods

### Study design

This retrospective bi-center study was waived by the ethics committee of Hannover Medical School. In all patients, PHP was regarded as the most appropriate therapy based on a local multidisciplinary tumor board decision. Contraindications for PHP included a recent history of transient ischemic attacks, heart failure with a left ventricular ejection fraction < 40%, hemorrhage-prone intracranial lesions, significant chronic obstructive or restrictive pulmonary disorder. ECOG performance status before PHP had to score 0 or 1.

All patients receiving PHP treatment either at Hannover Medical School between October 2014 and April 2021 (199 PHP in 93 patients) or at Asklepios Clinic Hamburg-Barmbek between April 2014 and April 2021 (57 PHP in 23 patients) were assessed for this study. The interventions were assigned to group_PROTAMINE_ (group_PRO_), group_REDUCED_PROTAMINE_ (group_REDPRO_) or group_NO_PROTAMINE_ (group_NOPRO_) according the intraprocedural coagulation management:

- Group_PRO_ included 92 patients treated with 192 PHP (Hannover Medical School: 75 patients/156 PHP; Asklepios Clinic Hamburg-Barmbek: 17 patients/36 PHP) that received full UFH reversal with protamine sulfate after PHP.
- Group_REDPRO_ contained 13 patients treated with 21 interventions at Asklepios Clinic Hamburg-Barmbek receiving a significantly reduced amount of protamine sulfate as UFH reversal following PHP.
- Group_NOPRO_ counted 43 interventions in 28 patients from Hannover Medical School with no UFH reversal after PHP.

Of note, 17 patients were included in both groups as they received protamine sulfate in the first PHP and reduced or no protamine sulfate in the following PHP.

### Procedures

**Fig. 1:**
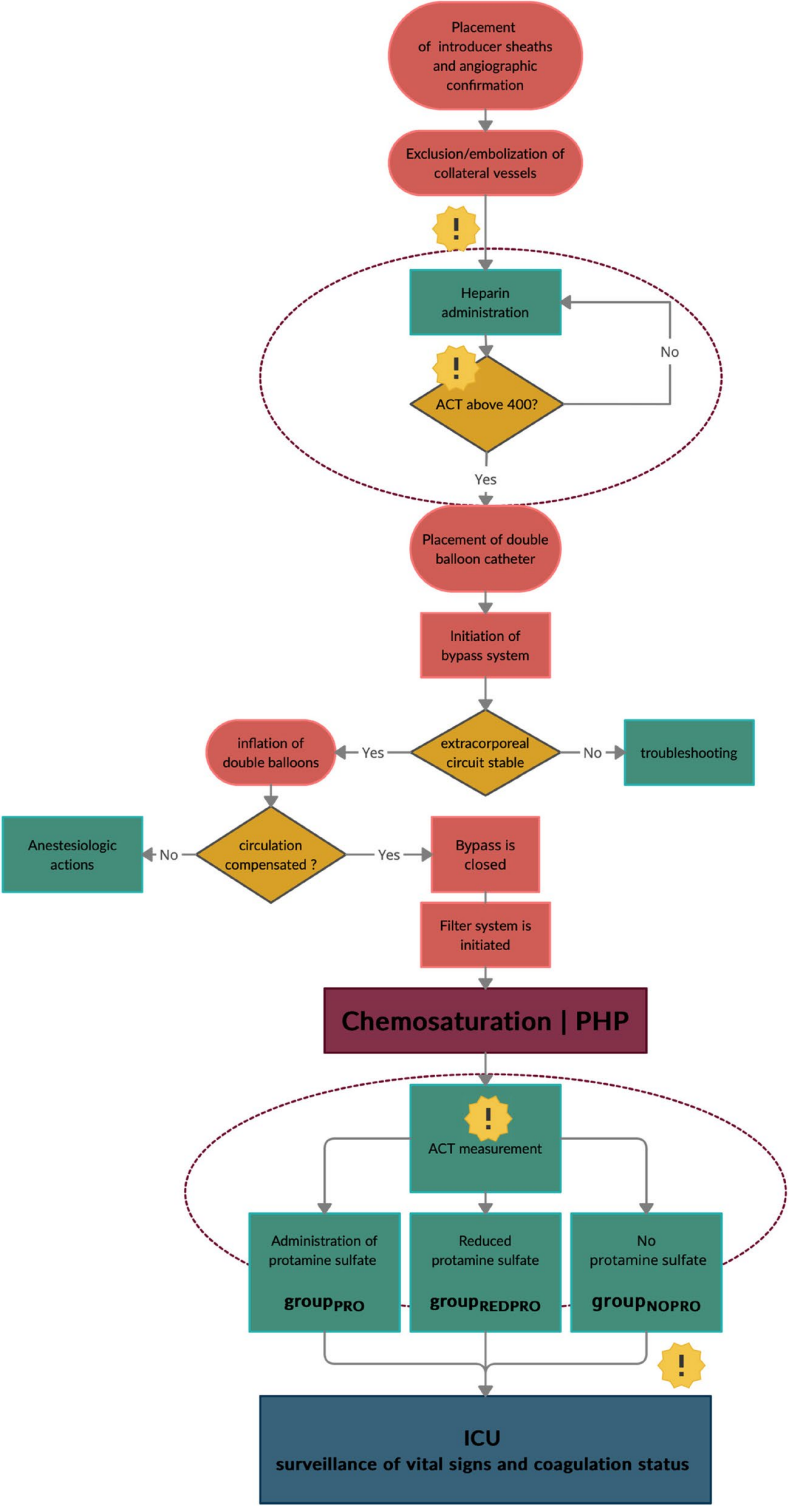
Flow chart of the PHP procedure. The dotted line marks the relevant steps for intraprocedural coagulation management. The exclamation marks point out the ACT measurements. *Of note*, ACT values were not always measured at fixed stages, repetitive measurements were performed at the discretion of the responsible staff but are excluded from this chart for overview reasons. PHP Percutaneous hepatic perfusion; ACT activated clotting time; ICU intensive care unit

All PHP procedures are performed in an angio suite under general anesthesia. As previously described [2, 7, 11, 22], a catheter is placed in the hepatic artery via a 4 or 5F sheath in the left common femoral artery. Introducer sheaths are also placed in the right common femoral vein (18F) and the right jugular vein (10F). Subsequently, UFH is administered as needed to achieve an ACT above 400s. Afterwards, the double balloon catheter, equipped with multiple fenestrations, is inserted through the femoral vein and placed in the hepatic segment of the IVC. The hemofiltration circuit is established: venous blood is aspirated through the double balloon catheter, flows through a veno-venous pump, initially passes the filters through a bypass line, and returns to the patient through the jugular return sheath. Next, the balloons are inflated close to the cavoatrial junction (cranial balloon) and in the subhepatic segment of the IVC (caudal balloon). Blood pressure regularly declines after occlusion of the IVC due to decreased cardiac inflow. After regeneration of the blood pressure, the bypass is closed and the melphalan specific filter system (containing two parallel filters), which separates up to 96% of melphalan from the venous blood [11, 23], is initiated. Now, the arterial chemoperfusion is started (wash-in phase). Dissolved in a 500 cc solution, the melphalan (2,5–3 mg/kg body weight) is infused in aliquots of each 100 cc at a rate of 0.4 ml/s. During the intervention, repeated ACT measurements and careful application of UFH (if necessary) provide a proper blood flow within the filtration system. Following the transarterial application of melphalan, the blood is still filtered for an additional 30 min (wash-out phase). At the end of the procedure, protamine sulfate was administered according to the initial UFH dose to antagonize the anticoagulant effect of the UFH in group_PRO_. In group_REDPRO_, a reduced dose of protamine sulfate was administered, aiming for antagonization of approximately two thirds of the active UFH. No UFH neutralization was performed in group_NOPRO._ Please refer to Fig. 1 for a flow chart outlining the procedural details.

After the chemosaturation, the introducer sheath in the left femoral artery was handled according to the immediate bleeding risk: in group_PRO_ and group_REDPRO_, the sheath was removed after administration of protamine sulfate (and the arterial puncture site was sealed using a vascular closure device; AngioSeal; Terumo, Japan), whereas in group_NOPRO_, the sheath remained in place. All patients were transferred to ICU, where the remaining sheaths were removed after normalization of the coagulation status, femoral compression devices were utilized to compress the puncture sites to support hemostasis.

Procedural data were retrospectively obtained from radiology and anesthesiology reports. ACT measurements were assigned to following stages (i) first ACT before heparin administration (ii) ACT before initiation of the extracorporeal circuit (iii) ACT after PHP (iv) last ACT value measured before transfer to ICU.

### Evaluation of peri-interventional complications

In both study centers, the periinterventional clinical reports, laboratory values and other PHP related findings were retrospectively assessed. The data were anonymized and shared for collaborative evaluation. For the analysis, the laboratory values of the first three postinterventional days were measured with the preprocedural baseline values. The Common Terminology Criteria for Adverse Events (CTCAEv5.0), which classifies the severity of adverse events into 5 levels (mild - death), was used to evaluate complications and adverse events. Major adverse cardiovascular events and peri-interventional mortality were analyzed [2].

### Analysis

Data were obtained retrospectively from medical records. Data are presented in relative and absolute values, mean or median (and interquartile range (IQR)). Continuous data were analyzed using Mann-Whitney-U test. A *p*-value of <0.05 was determined to be significant.

## Results

### Patient characteristics

Overall, 116 patients treated with 256 interventions (median 2 PHP per patient, range 1-8) were included. Group_PRO_ included 192 PHP treatments in 92 patients, group_REDPRO_ consisted of 21 PHP in 13 patients and group_NOPRO_ of 43 PHP in 28 patients. For detailed patient characteristics please refer to Table 1.

**Table 1:** Baseline patient characteristics of group_PRO_, group_REDPRO_ and group_NOPRO_

Parameter	Group_PRO_ (*n=*92 patients)		Group_REDPRO_ (*n=* 13 patients)		Group_NOPRO_ (*n=* 28 patients)	
Female; n; %	48	**52%**	6	**46%**	15	**54%**
Male; n; %	44	**48%**	7	**54%**	13	**46%**
Age (years); median; *interquartile range*	59	*52-66*	58	*51-66*	*58*	*50-63*
Malignancy; n; %						
• Uveal melanoma	57	**62%**	13	100%	14	**46%**
• Biliary tract cancer	17	**19%**	-	-	5	**17%**
• Hepatocellular carcinoma	6	**7%**	-	-	1	**4%**
• Mammary carcinoma	1	**1%**	-	-	5	**17%**
• Colorectal carcinoma	3	**3%**	-	-	1	**4%**
• NET carcinoma	2	**1%**	-	-	1	**4%**
• Pancreatic carcinoma	2	**2%**	-	-	-	-
• Periampullary carcinoma	3	**3%**	-	-	-	-
• Endometrial carcinoma	1	**1%**	-	-	-	-
• Soft tissue sarcoma	-	-	-	-	1	**4%**
Comorbidities; n; %						
• Hypertension	32	**35%**	3	23%	15	**54%**
• Diabetes mellitus	8	**9%**	3	23%	5	**18%**
• Thyroid disorder	13	**14%**	6	46%	3	**11%**
• Rhythm disorder	3	**2%**	2	15%	2	**4%**
• Secondary malignoma	2	**2%**	-	-	-	**-**
No. of PHP per patient; median; *min-max*	2	*1-8*	2	*1-4*	*2*	*1-8*
Treatments prior to PHP; n; %						
• Liver resection	14	**15%**	3	23%	5	**18%**
• Systemic chemotherapy	24	**26%**	1	8%	11	**39%**
• Surgery *and* systemic chemotherapy	6	**7%**	-	-	3	**11%**
• Immunotherapy	10	**11%**	2	15%	6	**21%**
• locoregional/intraarterial liver therapy other than PHP	16	**18%**	3	23%	6	**21%**
• No previous (liver directed) treatments	24	**26%**	4	31%	7	**25%**

*PHP* Percutaneous hepatic perfusion

### Procedural characteristics

Mean total UFH dose used for hemodilution was 41 059 I.E., 43 775 I.E. and 49 583 I.E. in group_PRO_, group_REDPRO_ and group_NOPRO_ respectively. Mean protamine sulfate used in group_PRO_ was 34 141 I.E., whereas in group_REDPRO_, 27 368 I.E (*p=*0.036). were used. For a detailed record of the procedural characteristics please refer to Table 2.

**Table 2:** Procedure characteristics of group_PRO_, group_REDPRO_ and group_NOPRO_

Parameter	Group_PRO_	Group_REDPRO_	Group_NOPRO_
Melphalan dose (mg; median (IQR)	179 (152-203)	200 (188-212)	184 (166-201)
Fluoroscopy time (mm; median (IQR))	7 (2-14)	12 (7-14)	6 (5-19)
Intervention time (hh:mm; median (IQR))	02:45 (02:28-03:07)	-	02:00 (01:54-02:15)
	Group_PRO vs NOPRO:_*p=*0.00001		
UFH Dose (I.U.; mean (IQR))	41 059 (32 125- 48 815)	43 775 (37 375- 46 000)	49 583 (40 000-60 000)
	group_PRO vs REDPRO:_*p=*0.368; group_PRO vs NOPRO:_*p=*0.0006; group_REDPRO vs NOPRO:_*p=*0.156		
Protamine sulfate (I.U.; mean (IQR))	34 141 (26 500- 40 000)	27 368 (20 000- 35 000)	-
	group_PRO vs REDPRO:_*p=*0.036		

Data of 97% of the interventions were available or this analysis

*IQR* Interquartile range, *UFH* Unfractionated heparin, *I.U* International unit

**Fig. 2:**
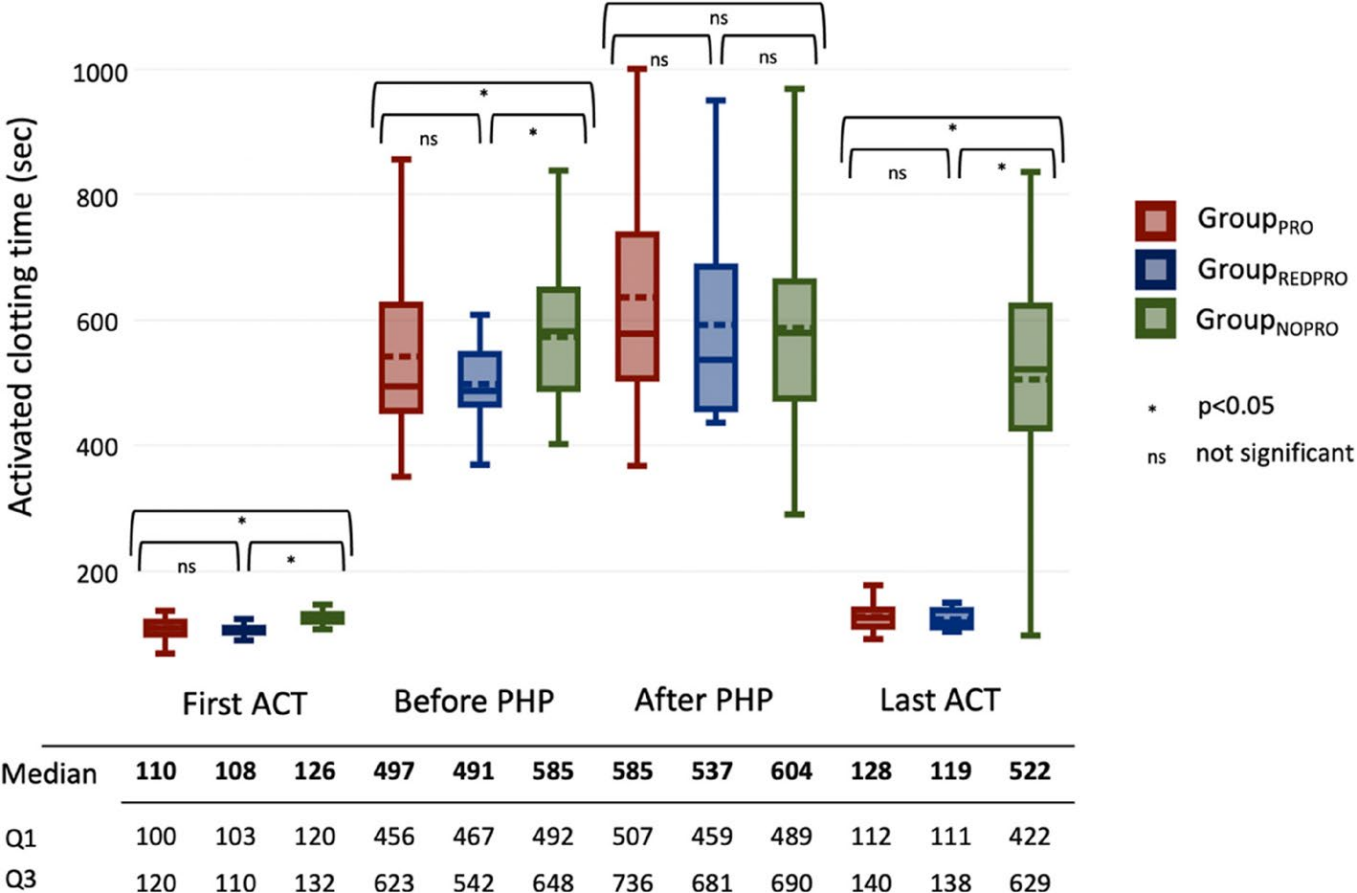
Boxplot diagram of the ACT values measured during PHP. 60% of the ACT values were available for this analysis. Different ACT measuring devices were used. PHP Percutaneous hepatic perfusion; ACT activated clotting time; Q1 first quartile; Q3 third quartile

Figure 2 displays the ACT values documented during the PHP procedures in all groups. As expected, the last ACT measured before transfer to ICU was significantly lower in group_PRO_ (median 128s) and group_REDPRO_ (median 119s) compared to group_NOPRO_ (median 522s; group_PRO_ vs _NOPRO:_*p=*0.00001; group_REDPRO_ vs _NOPRO:_*p=*0.00001). No major complications occurred during the PHP procedures. The median intervention time (measured from puncture until removal of the double balloon catheter/end of wash-out phase) was 2h 38m (2h 16m – 3h). The median time of hospitalization was 7 (6-10) days in groupPRO, 6 (5-7) days in groupREDPRO and 7 (5-10) in groupNOPRO.

### Periinterventional complications

#### Thromboembolic events

Group_PRO_: after 10/192 (5%) PHP procedures patients suffered from thromboembolic complications, six of which (3%) were major events (CTCAE grade 3-5):

- ◦ One patient developed an embolic occlusion of the anterior and middle cerebral artery approximately five hours after the fourth PHP procedure. Immediate percutaneous thrombectomy was performed. Tragically, the patient deceased after hemorrhagic conversion of the ischemic infarction with subsequent brain herniation.
- ◦ One patient presented with an occlusion of the left middle cerebral artery (MCA) and the distal right anterior cerebral artery approximately six hours following the first PHP procedure. Percutaneous recanalization and anticoagulation was immediately performed. Since the symptoms persisted, the patient was transferred to a neurologic rehabilitation clinic.
- ◦ One case of basilar artery thrombosis on the day after PHP, possibly due to de novo auricular fibrillations. The same patient developed deep vein thrombosis followed by pulmonary embolism.
- ◦ Central pulmonary embolism in one patient on the day after PHP.
- ◦ Another case of deep vein thrombosis, symptom onset (increasing leg swelling) starting a few days after PHP.
- ◦ One patient with a non-ST-segment elevation myocardial infarction (NSTEMI). Cardiac symptoms started on the evening after PHP.

Minor (CTCAE grade 1-2):

- ◦ On the morning after the fifth PHP, one patient with CCA presented with a transient hemiparesis. On cerebral MRI, a vascular, ischemic insult within the left-sided precentral cortex (most likely due to a thromboembolic event) was seen. The mild symptoms improved spontaneously, no lysis was performed.
- ◦ In total three cases of ischemic posterior cerebri artery (PCA) infarction were diagnosed on cross-sectional imaging. In all cases, lysis was not performed in view of the mild symptoms.

Group_REDPRO_: No (0%) thromboembolic events were recorded in group_PRO_.

Group_NOPRO_: No (0%) thromboembolic events were recorded in group_PRO_.

A detailed overview of the thromboembolic adverse events including treatment and sequelae (if present) is displayed in Table 3.

**Table 3:** Overview of thromboembolic (CTCAE grade 1-5) adverse events

Group	Adverse event	Area	Days after PHP	treatment	sequalae	*n*
group_PRO_	Ischemic stroke	MCA	1^st^	none	none	1
group_PRO_	Ischemic stroke	Bilateral PCA	4^th^	none	none	1
group_PRO_	Ischemic stroke	PCA	6^th^	none	none	1
group_PRO_	Ischemic stroke	PCA	5^th^	none	none	1
group_PRO_	Ischemic stroke; PE; DVT	Basilar artery (stroke)	2^nd^	Thrombectomy, anticoagulation	none	1
group_PRO_	Ischemic stroke	MCA and ACA	1^st^	Thrombectomy, anticoagulation	death	1
group_PRO_	Ischemic stroke	MCA (and ACA)	1^st^	Thrombectomy, anticoagulation	yes	1
group_PRO_	NSTEMI	LAD	1^st^	PTCA	none	1
group_PRO_	DVT	Femoral and popliteal vein	1^st^ week	Anticoagulation	none	1
group_PRO_	PE	Central pulmonary arteries	2^nd^	Anticoagulation	none	1

Day of the PHP procedure = 1^st^ day

*CTCAE* Common Terminology Criteria for Adverse Events, *PHP* Percutaneous hepatic perfusion, *MCA* Middle cerebral artery, *PCA* Posterior cerebral artery, *ACA* Anterior cerebral artery, *NSTEMI* Non-ST-segment elevation myocardial infarction, *LAD* Left anterior descending artery, *PTCA* Percutaneous transluminal coronary angioplasty, *DVT* Deep vein thrombosis, *PE* Pulmonary embolism

#### Hemorrhagic events

Group_PRO_: hemorrhagic events were recorded after 24/192 (13%) PHP, two of which (1%) were major (CTCAE grade 3-4):

- ◦ On the day of the PHP one patient developed a bleeding aneurysma spurium at the puncture site in the left groin with consecutive bleeding anemia (hemoglobin drop of two points). Surgical treatment was successful.
- ◦ One patient developed chronic ulcerous bleeding in the upper gastrointestinal tract 2 weeks after the second PHP. The ulcer was refractory to endoscopic and medical treatments and was treated surgically.

Minor (CTCAE grade 1-2) bleedings occurred after 22/192 interventions (12%) and included bleeding/hematoma at the puncture sites, vocal cord hematoma, intraocular bleeding, petechial bleeding, gastric ulceration and gastric and nasal mucosal bleedings (epistaxis) and bladder obstruction due to blood clots. Most hemorrhages occurred shortly after PHP.

Group_REDPRO_: no major (CTCAE grade 3-4) bleeding events occurred (0%). Minor (CTCAE grade 1-2) bleedings occurred after 2/21 interventions (10%).

Group_NOPRO_: following 12/43 (28%) PHP procedures, patients suffered from hemorrhagic events, one of which (2%) was CTCAE grade 4:

- ◦ The day after PHP, one patient developed an intracerebral bleeding in the right frontal lobe resulting in recurring convulsive syncope, dysarthria and dysphagia. The activated partial thromboplastin time at time of the event was 49 sec.. Unfortunately, despite intensive care treatment, the symptoms remained, the patient was discharged with a percutaneous endoscopic gastrostomy.

Minor events (CTCAE grade 1-2) were recorded after 11/43 PHP procedures (26%) including bleeding/hematoma at the puncture sites, and gastric and nasal mucosal bleedings, which were all self-limiting.

#### Toxicity and non-thromboembolic/non-hemorrhagic complications

Table 4 subsumes the rate of grade 3/4 thrombopenia, leucopenia and anemia following PHP. When comparing the post-interventional platetlet count of patients receiving protamine (group_PRO_ and group_REDPRO_) versus patients not receiving protamine (group_NOPRO_), a significant difference was found (*p=*0.00024).

**Table 4:** CTCAE grade 3 and 4 hematologic and hepatic toxicity after PHP in group_PRO_ (*n=*192), group_REDPRO_ (*n=*21) and group_NOPRO_ (*n=*43)

Adverse event	CTCAE Grade 3-4	
	Value	%
**Group**_**PRO**_		
Hematological toxicity		
- Thrombopenia	74	**39**
- Leucopenia	27	**14**
- Anemia	40	**21**
Hepatologic toxicity		
- AST increase	27	**14**
- ALT increase	19	**10**
- Hypoalbuminemia	6	**3**
- Hyperbilirubinemia	7	**4**
**Group**_**REDPRO**_		
Hematological toxicity		
- Thrombopenia	3	**14**
- Leucopenia	2	**10**
- Anemia	3	**14**
Hepatologic toxicity		
- AST increase	2	**10**
- ALT increase	1	**5**
- Hypoalbuminemia	-	**-**
- Hyperbilirubinemia	1	**5**
**Group**_**NOPRO**_		
**Hematological Toxicity**		
- Thrombopenia	10	**23**
- Leucopenia	4	**9**
- Anemia	8	**19**
**Hepatologic Toxicity**		
- AST increase	6	**14**
- ALT increase	1	**2**
- Hypoalbuminemia	4	**9**
- Hyperbilirubinemia	1	**2**

*CTCAE* Common Terminology Criteria for Adverse Events, *PHP* Percutaneous hepatic perfusion, *AST* Aspartate transaminase, *ALT* Alanine transaminase

In respect to liver toxicity, there was a significant increase of aminotransferases as markers of hepatic injury (Table 4).

In group_PRO_ one patient (1%) suffered from an anaphylactic shock after the administration of protamine. The allergic reaction was severe with hypotension, bronchospasm and skin and mucus membrane reactions. After immediate treatment, the symptoms resolved. Further complications are summarized in Table 5.

**Table 5:** CTCAE grade 3-5 complications following PHP

Adverse event *(treatment)*	Value	
	Grade 3/4	Grade 5
**Tacharrythmia absoluta***(cardioversion)*	2	
**Atrioventricular block**	1	
**Upper airway swelling***(cricothyrotomy)*	1	
**Tumor lysis syndrome**	2	
**Acute kidney injury**	1	
**SIRS or sepsis**	3	
**Death**		3

Generalized edema, ascites and/or pleural effusion due to overhydration and/or hypalbuminemia were treated with diuretics and paracentesis

*CTCAE* Common Terminology Criteria for Adverse Events, *PHP* percutaneous hepatic perfusion, *SIRS* Systemic inflammatory response syndrome

## Discussion

For chemosaturation with percutaneous hepatic perfusion (PHP), hemodilution with heparin is mandatory to ensure safe extracorporeal filtration. The results of this study indicate that UFH reversal with protamine sulfate after PHP might have an impact on both, post-interventional thromboembolic events and platelet count.

Hemorrhagic and thromboembolic events after PHP procedures performed with heparin reversal/protamine administration have been described in literature. In the landmark phase-III trial, Hughes et al. reported on one case of cerebral ischemia and myocardial infarction, each grade 3/4, within the first 72 hours after PHP [1]. Additional cases are described in retrospective studies: Within an analysis of 134 PHP in 51 patients, Karydis et al. documented two minor post-interventional cerebrovascular events (CTCAE 1/2) and five cases of cardiac ischemia (CTCAE 3/4) as well as seven mainly grade 3/4 thromboembolic events within two months after PHP (including two cases of pulmonary embolism, one each of IVC, left internal jugular, and vascular access site-related thrombus, and two lower limb DVTs) [4]. In another trial with 67 PHP in 35 patients, one case of peri-procedural cardiac ischemia and two patients with symptomatic pulmonary emboli, on the first and on the 17^th^ day after PHP were described [21].

Whereas the late post-interventional events (both in the mentioned literature as well as in our study cohort) are less likely a direct reaction to the administered protamine sulfate, a link between UFH reversal with protamine sulfate and the early periinterventional thromboembolic events is possible. However, other factors influencing thromboembolic events need to be considered. During cardiac surgery when cardiopulmonary bypass (CPB) is required, an acute systemic inflammatory response is provoked, which is mainly triggered by contact activation of blood by the artificial surfaces of the extracorporeal circuit [24]. This might as well be the case for the extracorporeal circuit in PHP. As coagulation and inflammation are closely linked through networks of both humoral and cellular components [25], their thrombogenic capacity needs to be discussed as a possible (additional) cause for thromboembolism. However, whereas all interventions in our study were performed using the extracorporeal circuit, only the interventions using full-dose protamine sulfate entailed thromboembolic events.

Concerning bleeding rates our analysis found more hemorrhagic events in group_NOPRO_ (28%) compared to group_PRO_ (13%). These rates are comparable to the current literature, where hemorrhagic events after PHP include minor (grade 1/2) bleedings (esp. at the puncture sites) in up to 30% [4, 21] as well as false aneurysms [26], haematemesis [26], epistaxis [21, 26], vaginal [21, 26] or abdominal [4] hemorrhage, disseminated intravascular coagulation [4], minor intracerebral bleeding [4] and retroperitoneal hematoma [27]. Since the rate of major bleeding events was similar with and without protamine sulfate, however, the higher risk of thromboembolic events might outweigh the risk of minor bleeding complications.

To date, the level of evidence for protamine dosing strategies is low and recommendations vary in the available guidelines [17, 19, 28]. Use and dosing of protamine sulfate are controversial and frequently based on local practices and individual experience rather than evidence. Boer et al. [17] outlined current protamine dosing strategies in cardiac surgery with CPB in a 2018 review [17]: in heparin dose-based protamine management the dosing ratio is either based on the initial heparin dose or the total heparin dose administered. Another approach is to administer protamine sulfate according to the concentration of heparin after the CPB [29]. The heparin concentration can be estimated based on mathematical or pharmacokinetic algorithms (model-based-titration) or by actual measurements of heparin concentration, anti-FIIa, or anti-FXa concentrations (measurement-based titration) [17]. There are several publications available on the effects of protamine titration on postoperative bleeding and transfusion in cardiac surgery, with conflicting results [17]. Since the use of protamine sulfate to antagonize UFH is complex and not standardized and side effects are common [16], we suggest that, given potentially desolate consequences, protamine sulfate should be handeld with care in a controlled PHP setting with ICU care afterwards.

While heparin - due to its reliability, availability and affordability - has found its way onto the World Health Organization’s list of essential drugs [30], its antidote protamine sulfate has a more difficult profile of side effects [31]. A systematic review of prospective and retrospective studies reporting serious anaphylactic reactions caused by protamine sulfate administration revealed an incidence of adverse reactions varying from 0.1% up to 11%^22^. In our study cohort, one case (1%) of severe anaphylaxis was observed right after the administration of protamine sulfate. Fortunately, in our patient, the allergic reaction was responsive to treatment, whereas other studies describe that the risk of in-hospital death is significantly increased in patients with adverse events after protamine administration [32].

Furthermore, we noticed a significantly lower rate of relevant post-interventional thrombopenia in the groups not receiving protamine sulfate compared to patients receiving protamine sulfate. Early and transient thrombopenia has been described in most PHP studies as a predominant side effect after PHP [1, 3, 4, 6, 21, 33–35]. The drop in platelet count has partly been attributed to the hematotoxicity of melphalan and to hematodilutive effects. Also, during the extracorporeal circuit, platelets can be activated, mechanically damaged and/or sequestrated in the microvasculature of the lungs e.g. [36]. Thus, a modest drop in platelet levels is almost inevitable after such procedures [37, 38]. Our results, however, indicate that the use of protamine sulfate might contribute more to the drop of platelets as so far recognized. Several studies reported significant thrombopenia when protamine was used to reverse heparin after cardiac surgery [17, 37, 38], suggesting that an immune-mediated reaction caused by protamine and heparin is involved in the pathophysiology [39]. More recent reports propose that protamine and heparin form multimolecular complexes following CPB that result in anti-protamine/heparin immunoglobulin G (IgG) class antibodies. A subset of these anti-protamine/heparin IgG antibodies activates platelets. So far, the consequence of the anti-protamine/heparin antibodies is unclear, but if these platelet-activating anti-protamine/heparin antibodies were already present at time of the heparin/protamine exposure (e.g., due to repetitive CPB/PHP), they might result in a more severe thrombopenia or even play a role in the development of thromboembolic events.

It should be kept in mind that PHP is a therapy approach for the palliative setting and even if a performance status ECOG 0 or 1 is a requirement for PHP treatment, the health status of our patients is fragile. Taking into account the potentially severe consequences of an anaphylaxis as well as the increased risk of both thromboembolism and thrombopenia, the necessity and value of UFH reversal with protamine sulfate should be assessed and adapted to the individual patient’s risk profile. One one hand, we regularely see patients who can be discharged from the ICU or intermediate care almost immediately after the procedure. In these patients, the administration of protamine can help as it reduces the time to ambulation and the time to hemostasis [40] without an increase in adverse events. The vast majority of patients present with moderate to severe frailty and are ususally kept in the ICU for one night. These patients might not benefit from agressive heparin reversal. Therefore, PHP patients should not be handled in a one-fits-all fashion.

A major limitation of this study is the retrospective study design, with all its potential confounders. Due to the retrospective nature, the study groups differ in size. Also, differences in ACT measurements due to the ACT measurement devices used should be considered. Furthermore, post-interventional blood samples may have been taken at different time points of the day. Due to the bicentric study design, inter-center differences might be underestimated. Most studies we used for comparisons were analyzing cardiac surgery with CPB, which clearly reduces the comparability. Previous reports on this specific matter are not available; thus, our data are rather *descriptive* and *hypothesis-generating* than conclusive, especially as in some cases the direct link between the coagulation management and the adverse event might be implied but cannot be proven.

## Conclusion

Our study implies that the use of protamine sulfate to reverse the hemodilutive effect of UFH after PHP might increase the risk of thromboembolic events as well as clinically significant post-interventional thrombocytopenia. Beyond that, protamine sulfate can provoke severe allergic reactions that potentially have an impact on the post-interventional mortality. In summary, we conclude that a paradigm shift regarding the standard use of protamine sulfate after PHP in low-risk patients without clinical bleeding signs should urgently be discussed. Certainly, appropriate after-care (e.g., on ICU) for patients receiving reduced or no protamine sulfate is necessary to ensure fast response in the unlikely event of significant bleeding complications.

## Data Availability

The datas analyzed during the current study are available from the corresponding author on reasonable request.

## Abbreviations

ACT: Activated clotting time
ALT: Alanine transaminase
AST: Aspartate transaminase
CPB: Cardiopulmonary bypass
CTCAE: Common Terminology Criteria for Adverse Events
DVT: Deep vein thrombosis
ICU: Intensive care unit
IgG: Immunoglobulin G
IQR: Interquartile range
IVC: Inferior vena cava
LAD: Left anterior descending artery
MCA: Middle cerebral artery
NSTEMI: Non-ST-segment elevation myocardial infarction
PCA: Posterior cerebri artery
PE: Pulmonary embolism
PHP: Percutaneous hepatic perfusion
PTCA: Percutaneous transluminal coronary angioplasty
SIRS: Systemic inflammatory response syndrome
UFH: Unfractionated heparin

## References

[1] Hughes MS, Zager J, Faries M, Alexander HR, Royal RE, Wood B (2016). Results of a Randomized Controlled Multicenter Phase III Trial of Percutaneous Hepatic Perfusion Compared with Best Available Care for Patients with Melanoma Liver Metastases. Ann Surg Oncol..

[2] Dewald CLA, Hinrichs JB, Becker LS, Maschke S, Meine TC, Saborowski A, et al. Chemosaturation with Percutaneous Hepatic Perfusion: Outcome and Safety in Patients with Metastasized Uveal Melanoma. Rofo. 2021;193(8):928–36. 10.1055/a-1348-1932 10.1055/a-1348-1932 33535258

[3] Artzner C, Mossakowski O, Hefferman G, Grosse U, Hoffmann R, Forschner A (2019). Chemosaturation with percutaneous hepatic perfusion of melphalan for liver-dominant metastatic uveal melanoma: a single center experience. Cancer Imaging..

[4] Karydis I, Gangi A, Wheater MJ, Choi J, Wilson I, Thomas K (2018). Percutaneous hepatic perfusion with melphalan in uveal melanoma: A safe and effective treatment modality in an orphan disease. J Surg Oncol..

[5] Kirstein MM, Marquardt S, Jedicke N, Marhenke S, Koppert W, Manns MP (2017). Safety and efficacy of chemosaturation in patients with primary and secondary liver tumors. J Cancer Res Clin Oncol..

[6] Estler A, Artzner C, Bitzer M, Nikolaou K, Hoffmann R, Hepp T (2021). Efficacy and tolerability of chemosaturation in patients with hepatic metastases from uveal melanoma. Acta Radiol..

[7] Dewald CLA, Warnke MM, Brüning R, Schneider MA, Wohlmuth P, Hinrichs JB (2021). Percutaneous Hepatic Perfusion (PHP) with Melphalan in Liver-Dominant Metastatic Uveal Melanoma: The German Experience. Cancers..

[8] Hinrichs JB, Wacker FK (2020). Lokoregionäre und lokal ablative Therapien von Lebertumoren. Internist..

[9] Dewald CLA, Becker LS, Maschke SK, Meine TC, Alten TA, Kirstein MM (2020). Percutaneous isolated hepatic perfusion (chemosaturation) with melphalan following right hemihepatectomy in patients with cholangiocarcinoma and metastatic uveal melanoma: peri- and post-interventional adverse events and therapy response compared to a matched group without prior liver surgery. Clin Exp Metastasis..

[10] Dewald CLA, Meine TC, Winther HMB, Kloeckner R, Maschke SK, Kirstein MM (2019). Chemosaturation Percutaneous Hepatic Perfusion (CS-PHP) with Melphalan: Evaluation of 2D-Perfusion Angiography (2D-PA) for Leakage Detection of the Venous Double-Balloon Catheter. Cardiovasc Intervent Radiol..

[11] Vogel A, Gupta S, Zeile M, von Haken R, Brüning R, Lotz G (2016). Chemosaturation Percutaneous Hepatic Perfusion: A Systematic Review. Adv Ther..

[12] Hirsh J, Warkentin TE, Shaughnessy SG, Anand SS, Halperin JL, Raschke R (2001). Heparin and Low-Molecular-Weight Heparin Mechanisms of Action, Pharmacokinetics, Dosing, Monitoring, Efficacy, and Safety. Chest..

[13] Hirsh J, Anand SS, Halperin JL, Fuster V (2001). Mechanism of Action and Pharmacology of Unfractionated Heparin. Arterioscler Thromb Vasc Biol.

[14] Ku MJ, Kim SW, Lee S, Chang JW, Lee J (2020). Risk Factors Associated with Difficult Reversal of Heparin by Protamine Sulfate in Cardiopulmonary Bypass: An Ignored Issue. Korean J Thorac Cardiovasc Surg..

[15] Delcath Systems, Inc (2014). DELCATH HEPATIC CHEMOSAT® DELIVERY SYSTEM.

[16] Sokolowska E, Kalaska B, Miklosz J, Mogielnicki A (2016). The toxicology of heparin reversal with protamine: past, present and future. Expert Opin Drug Metab Toxicol.

[17] Boer C, Meesters MI, Veerhoek D, Vonk ABA (2018). Anticoagulant and side-effects of protamine in cardiac surgery: a narrative review. Br J Anaesth.

[18] Portmann AF, Holden WD. PROTAMINE (SALMINE) SULPHATE, HEPARIN, AND BLOOD COAGULATION. :8. 10.1172/JCI102210 PMC439701 15395947

[19] Pagano D, Milojevic M, Meesters MI, Benedetto U, Bolliger D, von Heymann C (2018). 2017 EACTS/EACTA Guidelines on patient blood management for adult cardiac surgery. Eur J CardioThorac Surg.

[20] Chandiramani AS, Jenkin I, Botezatu B, Harky A. Protamine-Induced Coronary Graft Thrombosis: A Review. Journal of Cardiothoracic and Vascular Anesthesia. 2021;S1053077021008910. 10.1053/j.jvca.2021.10.008 34774407

[21] Meijer TS, Burgmans MC, Fiocco M, de Geus-Oei LF, Kapiteijn E, de Leede EM (2019). Safety of Percutaneous Hepatic Perfusion with Melphalan in Patients with Unresectable Liver Metastases from Ocular Melanoma Using the Delcath Systems’ Second-Generation Hemofiltration System: A Prospective Non-randomized Phase II Trial. Cardiovasc Intervent Radiol..

[22] Schönfeld L, Hinrichs JB, Marquardt S, Voigtländer T, Dewald C, Koppert W (2020). Chemosaturation with percutaneous hepatic perfusion is effective in patients with ocular melanoma and cholangiocarcinoma. J Cancer Res Clin Oncol..

[23] de Leede EM, Burgmans MC, Meijer TS, Martini CH, Tijl FGJ, Vuyk J (2017). Prospective Clinical and Pharmacological Evaluation of the Delcath System’s Second-Generation (GEN2) Hemofiltration System in Patients Undergoing Percutaneous Hepatic Perfusion with Melphalan. Cardiovasc Intervent Radiol..

[24] Day JRS, Taylor KM (2005). The systemic inflammatory response syndrome and cardiopulmonary bypass. Int J Surg.

[25] Levy JH, Tanaka KA (2003). Inflammatory response to cardiopulmonary bypass. Ann Thorac Surg.

[26] Vogl TJ, Koch SA, Lotz G, Gebauer B, Willinek W, Engelke C (2017). Percutaneous Isolated Hepatic Perfusion as a Treatment for Isolated Hepatic Metastases of Uveal Melanoma: Patient Outcome and Safety in a Multi-centre Study. Cardiovasc Intervent Radiol..

[27] Vogl TJ, Zangos S, Scholtz JE, Schmitt F, Paetzold S, Trojan J (2014). Chemosaturation with Percutaneous Hepatic Perfusions of Melphalan for Hepatic Metastases: Experience from Two European Centers. Rofo..

[28] Ferraris VA, Brown JR, Despotis GJ, Hammon JW, Reece TB, Saha SP (2011). 2011 Update to The Society of Thoracic Surgeons and the Society of Cardiovascular Anesthesiologists Blood Conservation Clinical Practice Guidelines**The International Consortium for Evidence Based Perfusion formally endorses these guidelines. Ann Thorac Surg.

[29] Koster A, Börgermann J, Gummert J, Rudloff M, Zittermann A, Schirmer U (2014). Protamine Overdose and Its Impact on Coagulation, Bleeding, and Transfusions After Cardiopulmonary Bypass: Results of a Randomized Double-Blind Controlled Pilot Study. Clin Appl Thromb Hemost..

[30] 22th WHO Model List of Essential Medicines (September 2021) https://www.who.int/publications/i/item/WHO-MHP-HPS-EML-2021.02.

[31] Valchanov K, Falter F, George S, Burt C, Roscoe A, Ng C (2019). Three Cases of Anaphylaxis to Protamine: Management of Anticoagulation Reversal. J Cardiothorac Vasc Anesth.

[32] Kimmel SE, Sekeres M, Berlin JA, Ellison N (2002). Mortality and Adverse Events After Protamine Administration in Patients Undergoing Cardiopulmonary Bypass. Anesth Analg.

[33] Brüning R, Tiede M, Schneider M, Wohlmuth P, Weilert H, Oldhafer K (2020). Unresectable Hepatic Metastasis of Uveal Melanoma: Hepatic Chemosaturation with High-Dose Melphalan—Long-Term Overall Survival Negatively Correlates with Tumor Burden. Radiol Res Pract..

[34] Struck MF, Kliem P, Ebel S, Bauer A, Gössmann H, Veelken R (2021). Percutaneous hepatic melphalan perfusion: Single center experience of procedural characteristics, hemody-namic response, complications, and postoperative recovery. Chen RJ, editor. PLoS One..

[35] Pingpank JF, Libutti SK, Chang R, Wood BJ, Neeman Z, Kam AW (2005). Phase I Study of Hepatic Arterial Melphalan Infusion and Hepatic Venous Hemofiltration Using Percutaneously Placed Catheters in Patients With Unresectable Hepatic Malignancies. J Clin Oncol..

[36] Nathan N, Mercury P, Denizot Y, Cornu E, Laskar M, Arnoux B (1994). Effects of the Platelet-Activating Factor Receptor Antagonist BN 52021 on Hematologic Variables and Blood Loss During and After Cardiopulmonary Bypass. Anesth Analg.

[37] Al-Mondhiry H, Pierce WS, Basarab RM (1985). Protamine-Induced Thrombocytopenia and Leukopenia. Thromb Haemost..

[38] Singla A, Sullivan MJ, Lee G, Bartholomew J, Kapadia S, Aster RH, et al. Protamine-induced immune thrombocytopenia: Protamine-Induced Immune Thrombocytopenia. Transfusion. 2013 Mar;n/a-n/a. 10.1111/trf.12112 PMC3655112 23384227

[39] Bakchoul T, Jouni R, Warkentin TE (2016). Protamine (heparin)-induced thrombocytopenia: a review of the serological and clinical features associated with anti-protamine/heparin antibodies. J Thromb Haemost..

[40] Kewcharoen J, Shah K, Bhardwaj R, Contractor T, Turagam MK, Mandapati R (2022). Periprocedural outcomes of protamine administration after catheter ablation of atrial fibrillation. Rev Cardiovasc Med..

